# Inequities in prenatal neonatology consultation in high-mortality neonatal populations

**DOI:** 10.1038/s41372-025-02377-z

**Published:** 2025-08-20

**Authors:** Samantha L. Simpson, Kylie Mena, DonnaMaria E. Cortezzo, Chunyan Liu, Shelley R. Ehrlich, Sarah Eaton, Ting Ting Fu, Andrew F. Beck, James M. Greenberg, Emily R. Miller

**Affiliations:** 1 Cincinnati Children’s Hospital Medical Center, Division of Neonatal and Pulmonary Biology, Cincinnati, OH USA; 2 Cincinnati Children’s Hospital Medical Center, Office of Population Health, Cincinnati, OH USA; 3 Cincinnati Children’s Hospital Medical Center, Michael A. Fisher Child Health Equity Center, Cincinnati, OH USA; 4 University of Cincinnati College of Medicine, Department of Pediatrics, Cincinnati, OH USA; 5 University of Colorado School of Medicine, Department of Pediatrics, Section of Neonatology, Aurora, CO USA; 6 Cincinnati Children’s Hospital Medical Center, Division of Pain and Palliative Medicine, Cincinnati, OH USA; 7 University of Cincinnati College of Medicine, Department of Anesthesiology, Cincinnati, OH USA; 8 Connecticut Children’s Medical Center, Division of Neonatology, Hartford, CT USA; 9 Connecticut Children’s Medical Center, Division of Pain and Palliative Care, Hartford, CT USA; 10 Connecticut Children’s Medical Center, Fetal Care Program, Hartford, CT USA; 11 University of Connecticut School of Medicine, Department of Pediatrics, Farmington, CT USA; 12 Cincinnati Children’s Hospital Medical Center, Division of Biostatistics and Epidemiology, Cincinnati, OH USA; 13 University of Cincinnati, College of Medicine, Department of Environmental and Public Health Sciences, Cincinnati, OH USA; 14 Cincinnati Children’s Hospital Medical Center, Division of General and Community Pediatrics, Cincinnati, OH USA; 15 Cincinnati Children’s Hospital Medical Center, Division of Hospital Medicine, Cincinnati, OH USA

**Keywords:** Paediatrics, Health services

## Abstract

**Objectives:**

To explore inequities in prenatal consultation and parental resuscitation decisions across high-mortality conditions.

**Study design:**

We conducted a retrospective chart review of pregnant people whose liveborn neonates were diagnosed with high-mortality conditions. We examined two cohorts: periviable infants (22 0/7–24 6/7 weeks) and infants with severe congenital anomalies.

**Results:**

A total of 194 neonates met eligibility criteria for the periviable cohort, 197 for the congenital anomaly cohort. In the periviable cohort, 94% of White vs. 81% of Black pregnant people received neonatology consultation (*p* = 0.009). A total of 96% of those with commercial insurance vs. 82% of those with Medicaid received consultation (*p* = 0.005). Half of Hispanic pregnant people did not receive neonatology consultation (*p* = 0.02). In the congenital anomaly cohort, pregnant people who spoke a language other than English were less likely to receive consultation (44% vs. 81%, *p* = 0.02).

**Conclusions:**

This regional assessment found previously unrecognized inequities in prenatal neonatology consultation.

## Introduction

Despite a reduction in neonatal mortality over the last several decades [1], multiple conditions continue to confer a high likelihood of death, including birth during the periviable period (22–24 weeks gestation) and birth with certain congenital anomalies. Neurodevelopmental outcomes, perceived quality-of-life, and risk of mortality with these cases can be difficult to predict prenatally. Thus, either invasive interventions or comfort measures may be ethically and legally permissible [2]. It is imperative that clinical teams and families communicate prenatally to develop the most appropriate care plan for the birth and potentially, the end of life.

Inequities in pediatric end-of-life care are influenced by social determinants of health (SDoH), defined as “the conditions in which people are born, grow, work, live, and age, and the wider set of forces and systems shaping the conditions of daily life” [3–12]. Such conditions can include the lived experiences of systemic racism. As such, Black, Hispanic, and low-income children receive less palliative care and more high-intensity end-of-life medical interventions (such as cardiopulmonary resuscitation, intubation, and intensive care unit admission) than their White peers and those with moderate/high income.

Data is more limited in the perinatal period. Some existing studies, however, suggest increased odds of high-intensity resuscitation and interventions for periviable infants born to Black and Hispanic pregnant people and for those with public insurance or low educational attainment [13, 14]. More recent and comprehensive national data, however, showed that non-White periviable infants were less likely than White infants to receive active treatment after delivery [15]. Another study investigated parental preferences for delivery room resuscitation for multiple high-risk neonatal populations, illustrating that few parents recalled discussing delivery room resuscitation preferences and noting that White pregnant people were more likely than non-White individuals to recall discussing options for comfort care [16]. Still, published rates of prenatal consultation for periviable births and several life-limiting fetal diagnoses are low [17–19]. Moreover, to our knowledge, no studies have rigorously interrogated how SDoH and sociodemographic characteristics may impact prenatal consultation.

Inequities in neonatology consultation and counseling may perpetuate inequities in resuscitation by race, ethnicity, or socioeconomic status. In this study, we aimed to assess the relationships of SDoH, race, ethnicity, language, and clinical factors with prenatal neonatology consultation and parental decisions for resuscitation for fetal diagnoses at high risk of early neonatal mortality. Specifically, we sought to compare markers of the SDoH and sociodemographic characteristics of patients who received a prenatal neonatology consultation with those who did not, determine associations with parental delivery room resuscitation decisions, and evaluate consultation patterns at various delivery locations. We hypothesized that prenatal neonatology consultations would occur less often for marginalized, disadvantaged populations, potentially influencing resuscitation decision making.

## Methods

### Study design, study population, and data source

We conducted an Institutional Review Board-approved retrospective chart review of pregnant people with eligible high-mortality fetal diagnoses who delivered at 1 of 5 regional specialty perinatal centers between January 1, 2015, and December 31, 2021. Eligible conditions were identified by literature review [20] and expert opinion (Supplementary Table 1). Conditions included birth in the periviable period (22 0/7–24 6/7 weeks gestation), genetic diagnoses (e.g., trisomy 13, trisomy 18), and congenital anomalies (e.g., giant encephalocele, bilateral renal agenesis). Consensus of authors with expertise in neonatology and palliative care resolved uncertainties regarding eligibility. Conditions were dichotomized into two analysis cohorts: a periviable cohort and a congenital anomaly cohort to account for differential duration between consultation and delivery and parental expectations regarding prognosis. We excluded pregnant people whose pregnancies did not result in a livebirth and those who were only offered comfort measures.

### Outcome measures, exposures, and covariates

All data were obtained from the electronic health record. The primary outcome was prenatal inpatient or outpatient neonatology consultation. The secondary outcome was documented parental decision for neonatal delivery room resuscitation, categorized as full active resuscitation, limited resuscitation, or comfort care.

Primary exposures included several sociodemographic characteristics and markers of the SDoH. Self-reported race and ethnicity served as proxies for structural and interpersonal racism [21, 22]. Demographic variables were treated as binary: race (White vs. non-White to encapsulate anti-Black and anti-Brown racism), ethnicity (Hispanic vs. non-Hispanic), and payor type (Medicaid/self-pay vs. commercial insurance). To further define socioeconomic context, we geocoded each home address, enabling linkage to socioeconomic data available from the U.S. Census. Specifically, we joined each address to census tract-level data, including a community material deprivation index [23–26].

Additional exposures included relevant clinical characteristics [13, 14, 27]. Adequacy of prenatal obstetrical care was defined by the obstetric provider as the following: the first prenatal visit occurred within the first 4 months of pregnancy, and the pregnant person attended 80% of the recommended visits. Delivery location was identified to assess regional variation in care. Five regional specialty perinatal centers were selected to encapsulate a range of care delivery sites. This included academic medical centers (a level II nursery and a level III neonatal intensive care unit (NICU)), private healthcare facilities (a level II nursery and a level III NICU), and a quaternary pediatric medical and surgical referral center (level IV NICU). The time between admission, consultation, and delivery were obtained by time stamps in the electronic health record. If multiple consultations occurred, the first consultation time was utilized.

### Statistical analyses

Analyses for periviable cohort and congenital anomaly cohort were done separately. For both cohorts, we used descriptive statistics to summarize sociodemographic, contextual, and clinical characteristics in pregnant people who did or did not receive a prenatal consult. Chi-square or Fisher’s exact tests were used to compare the percentages of categorical characteristics. Non-parametric Wilcoxon rank sum or Kruskal–Wallis tests were used to compare medians for continuous variables.

We performed multivariable modeling using logistic regression to examine the association between pre-selected characteristics (including *race* and *insurance*) and *receiving a prenatal consultation* (outcome of interest). For the periviable cohort, variables including the *duration between admission and delivery* and *adequacy of prenatal care* were considered as potential effect modifiers and their effects were tested through their interactions with race and with insurance. For this cohort, language was not selected as a covariate due to very few families speaking a language other than English. For the congenital anomaly cohort, adequacy of prenatal care and preferred language (pending sample size) were considered as potential effect modifiers. The timing between admission and delivery was not considered clinically relevant for the congenital anomaly cohort. Backward variable selection using *p* < 0.1 provided us final model components. Site was first pursued as a random effect to accommodate the cluster effect. However, considering the number of sites was small, and the sample size for some sites was very small, we recognized the possibility of convergence issues. When non-convergence happened, site was used as a fixed effect and subject to variable selection.

To visually assess the relationships between continuous variables (such as the deprivation index and timing of admission to delivery) and prenatal consultation, we ran generalized additive models (GAM) to allow for evaluation of nonlinear relationships through the application of smoothing parameters.

Three patients belonged to both the periviable and congenital anomaly cohorts. Sensitivity analyses were conducted, excluding these patients from both cohorts. A separate sensitivity analysis was done for the congenital anomaly cohort where fetuses with only cardiac diagnoses were excluded.

Decision for resuscitation in the delivery room was evaluated both as an infant-level analysis (as families may make different decisions for each infant) and a pregnant person-level analysis (to not overrepresent pregnant people who may be represented more than once within the dataset and who may make congruent decisions). One pregnant person made incongruent resuscitation decisions for their twins; they were excluded from pregnant person-level analysis but not infant-level analysis.

A *p* < 0.05 was considered statistically significant. SAS 9.4 and R 4.2.2 software were used for analysis. The R package “mgcv” version 1.8-41 was used for GAM analysis.

## Results

**Fig. 1:**
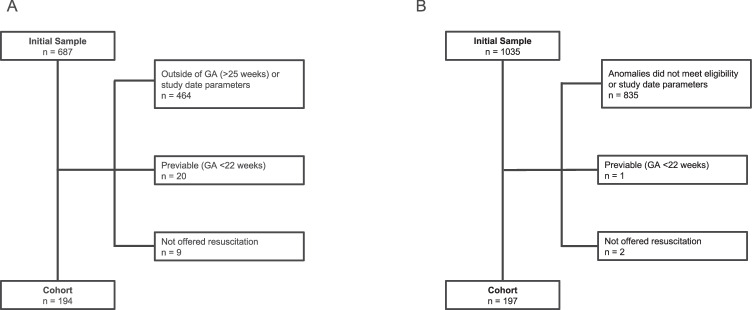
Flow diagram of infant selection. Panel **A** depicts the periviable cohort. Panel **B** depicts the congenital anomaly cohort.

Over 1700 charts were screened with 194 neonates meeting inclusion criteria for the periviable cohort and 197 for the congenital anomaly cohort (Fig. 1). Three neonates met criteria for both cohorts: they were born in the periviable period, 1 each with hydrops, trisomy 18, and a high-risk sacrococcygeal teratoma.

### Periviable cohort

There were 171 pregnant people for the 194 neonates in the periviable cohort. One pregnant person with 2 periviable births during the study period was included twice. This cohort had a median age of 28.8 years and identified as 51% White, 97% non-Hispanic, and 95% English-speaking (Table 1). A slight majority had Medicaid insurance or were self-pay (57%). The median gestational age at delivery was 24 0/7 weeks. Most received a neonatology consultation prior to delivery (88%) with a median time from admission to consult of 3.6 h and from consult to delivery of 66.3 h. Most (83%) chose full active resuscitation after delivery.

**Table 1:** Descriptive table of pregnant people by prenatal consult among periviable cohort.

Variable	Overall (*N* = 172)	Did not receive a consult (*N* = 21)	Received a consult (*N* = 151)	*p* value^a^
**Demographics**				
Race				0.0187
White	87 (50.6%)	5 (5.7%)	82 (94.3%)	
Black or African American	73 (42.4%)	14 (19.2%)	59 (80.8%)	
American Indian or Alaska native	1 (0.6%)		1 (100%)	
Asian	4 (2.3%)		4 (100%)	
Native Hawaiian or Other Pacific Islander	1 (0.6%)	1 (100%)		
Multiracial/Unknown	6 (3.5%)	1 (16.7%)	5 (83.3%)	
Dichotomized race				0.0088
White	87 (50.6%)	5 (5.7%)	82 (94.3%)	
Non-White	85 (49.4%)	16 (18.8%)	69 (81.2%)	
Ethnicity				0.0249
Non-Hispanic	166 (96.5%)	18 (10.8%)	148 (89.2%)	
Hispanic	6 (3.5%)	3 (50.0%)	3 (50.0%)	
Preferred language				0.3022
English	163 (94.8%)	19 (11.7%)	144 (88.3%)	
Spanish	9 (5.2%)	2 (22.2%)	7 (77.8%)	
Age at delivery (years)				0.0570
Median (Q1, Q3)	28.8 (24.7, 33.8)	26.1 (19.1, 30.2)	29.1 (24.9, 34.1)	
Insurance payor type				0.0045
Medicaid or self-pay	98 (57.0%)	18 (18.4%)	80 (81.6%)	
Commercial	74 (43.0%)	3 (4.1%)	71 (95.9%)	
Community deprivation index				0.1197
Median (Q1, Q3)	0.3 (0.2, 0.4)	0.4 (0.2, 0.6)	0.3 (0.2, 0.4)^b^	
**Prenatal care**				
Fertility treatment				0.3637
No	161 (93.6%)	21 (13.0%)	140 (87.0%)	
Yes	11 (6.4%)		11 (100.0%)	
Prenatal care				0.0003
Good	149 (86.6%)	12 (8.1%)	137 (91.9%)	
Inadequate (None, Scant, or Late)	23 (13.4%)	9 (39.1%)	14 (60.9%)	
Birth hospital				0.0001
Two academic medical centers (Level IV and Level III)	78 (45.3%)	6 (7.7%)	72 (92.3%)	
One private level III NICU	88 (51.2%)	11 (12.5%)	77 (87.5%)	
Two level II NICUs	6 (3.5%)	4 (66.7%)	2 (33.3%)	
**Outcomes**				
Infant gestational age at birth (weeks)				0.4744
Median (Q1, Q3)	24.0 (23.4, 24.6)	24.0 (23.1, 24.4)	24.1 (23.4, 24.6)	
Timing: admission to consult (h)				–
Median (Q1, Q3)	3.6 (0.9, 17.2)		3.6 (0.9, 17.2)	
Timing: consult to birth (h)				–
Median (Q1, Q3)	66.3 (18.2, 150.5)		66.3 (18.2, 150.5)	
Timing: admission to birth (h)				
Median (Q1, Q3)	51.3 (7.3, 117.8)	0.8 (0.1, 2.0)	69.6 (18.2, 138.6)	<0.0001
Decision for resuscitation^c^				0.0398
Full active resuscitation	140 (81.9%)	20 (14.3%)	120 (85.7%)	
Limited resuscitation	27 (15.8%)	0 (0.0%)	27 (100.0%)	
Comfort care	4 (2.3%)	1 (25.0%)	3 (75.0%)	

^a^Wilcoxon rank sum test for continuous variables and Chi-square or Fisher’s exact test for categorical variables.

^b^*n* = 148 given inability to geocode available address information.

^c^*n* = 171 as 1 pregnant individual who made different resuscitation decisions between twins was excluded.

**Fig. 2:**
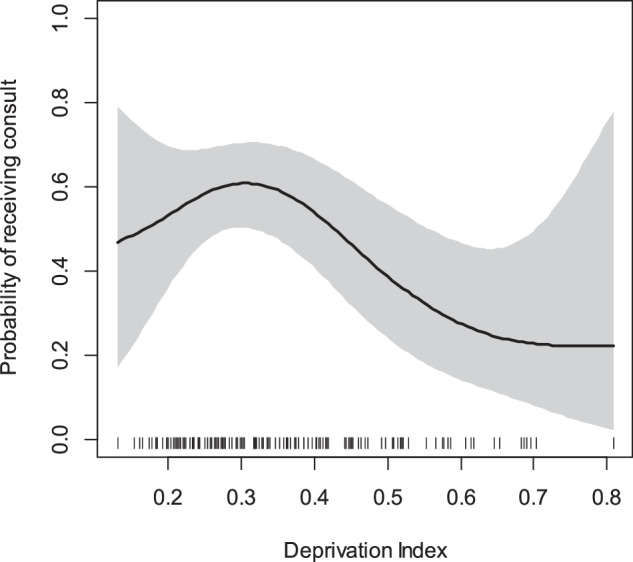
Probability of receiving prenatal consultation with neonatology by deprivation index for the periviable cohort.

A total of 94% of White vs. 81% of non-White pregnant people received prenatal neonatology consultation (*p* = 0.009). Two-thirds (67%) of those who did not receive consultation were Black. Half of Hispanic pregnant people did not receive consultation (*p* = 0.02). Commercially-insured pregnant people were also more likely to receive consultation compared to those with Medicaid/self-pay (96% vs. 82%, *p* = 0.005) (Table 1). While there was not a statistically significant difference in the median census tract-level deprivation index score between prenatal consultation groups in bivariate analysis, the non-linear curve generated by GAM showed the probability of receiving prenatal neonatology consultation had a decreasing trend as the deprivation index increased beyond 0.3, which is the median of the deprivation index across national tracts (Fig. 2) [28].

The median time between admission and delivery was significantly shorter in those who did not receive consultation compared to those who did: 0.8 h vs. 69.6 h (Table 1, *p* < 0.001). The median time between admission and delivery did not differ statistically by race or payor type (60.2 h for White pregnant people vs. 47.8 h for non-White pregnant people [*p* = 0.454], 54.8 h for those with commercial insurance vs. 50.8 h for those with Medicaid/self-pay [*p* = 0.17]).

Adequate prenatal care, as defined above, differed between those who received consultation and those who did not (91% vs. 57%, *p* < 0.001, Table 1). There were inequities in adequate prenatal care by race and payor type (95% of White pregnant people vs. 78% of non-White [*p* < 0.001]; 96% with commercial insurance vs. 80% with Medicaid insurance or self-pay [*p* = 0.002]).

**Fig. 3:**
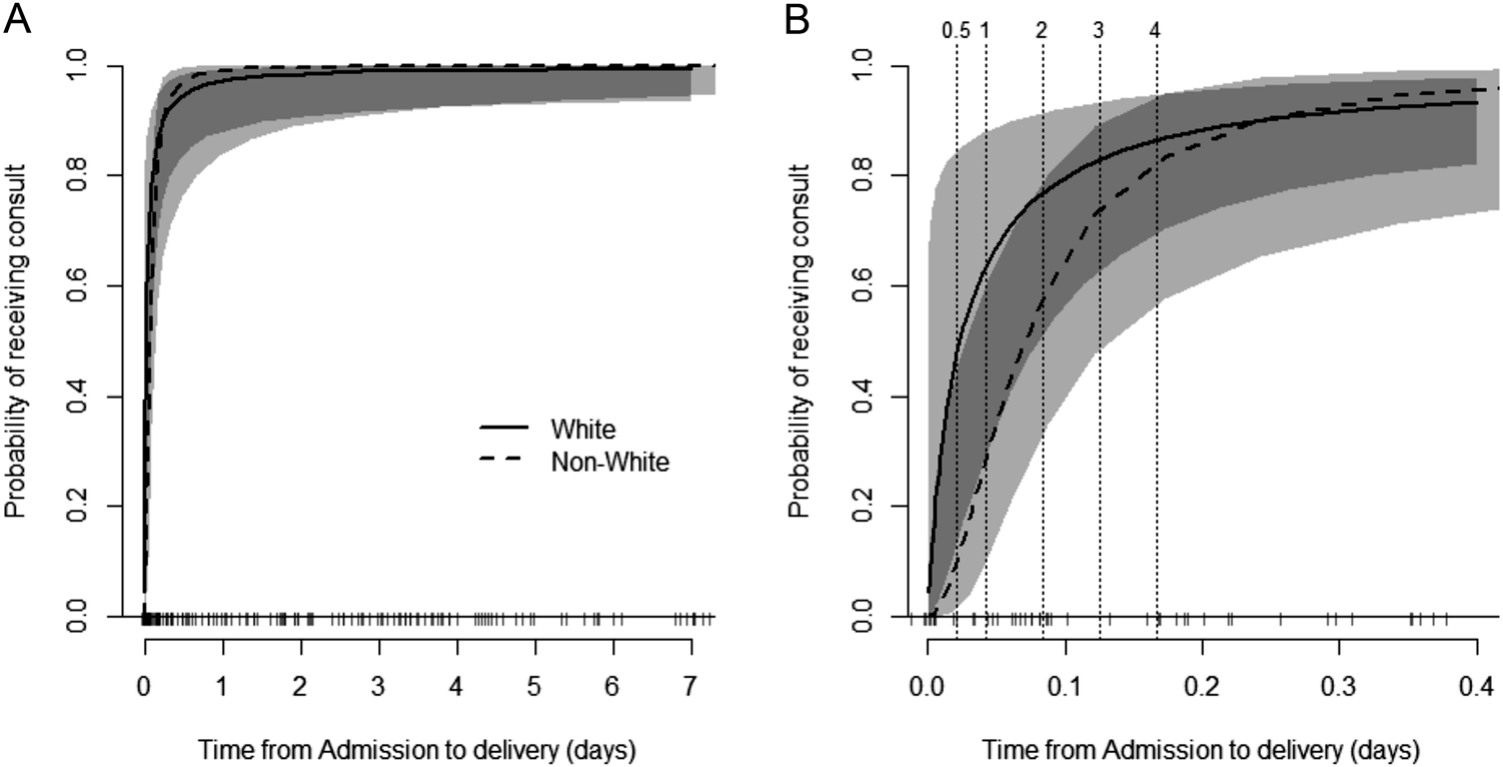
Impact of time from admission to delivery (days) on the probability of receiving prenatal consultation in the periviable cohort using GAM. The time between admission and delivery only affect the chance of receiving consult when its value is small as shown in (**A**). **B** is a zoomed-in version of the left panel where *x*-axis only ranged from 0 to 0.4 days. The vertical dashed lines correspond to ½, 1, 2, 3, 4 h after admission.

In the multivariable analysis, backward model selection was performed as described above. Site of delivery caused non-convergence when used as a random effect and was not significant as a fixed effect. Prenatal care was not significant as a main effect or as an effect modifier. In the final model, timing of admission to delivery modified the relationship between prenatal consult and race (interaction term *p* = 0.04, Table 2) but not prenatal consultation and insurance (interaction term *p* = 0.59, Supplementary Table 2). The probability of receiving a prenatal consultation for patients who delivered soon after admission (i.e., within 3–4 h of admission) was consistently higher among White pregnant people as compared to Non-White pregnant people. White pregnant people who delivered within the first hour of admission had an almost 7-fold increased odds of receiving a prenatal consult than Non-White pregnant people. For deliveries occurring 3 h after admission, White pregnant people had an increased, but more attenuated, 2.4-fold increased odds of having a prenatal consult compared Non-White pregnant people (Table 2 and Fig. 3). Those with commercial insurance had a higher odds of receiving prenatal consultation than those with Medicaid or self-pay and was marginally significant (OR [95%CI] = 4.31 [0.91, 20.36], *p* = 0.065). Additional models tested are included in Supplementary Table 2.

**Table 2:** Multivariable modeling investigating factors affecting probability of prenatal consult for pregnant people in the analysis cohorts.

Model	Effect	Contrast	Odds ratio	*p* value
Periviable cohort (*N* = 172)^a^				
1	Race of pregnant person	White vs. non-White	–	0.0092
	Time from admission to delivery (h)		–	0.0096
	Time from admission to delivery by race of pregnant person interaction	White vs. Non-White | Time from admission to delivery^b^ = 1 h	6.88 (1.38, 34.20)	0.0403
		White vs. Non-White | Time from admission to delivery^b^ = 3 h	2.44 (0.51, 11.76)	
Insurance		Commercial vs. Medicaid/Self-pay	4.31 (0.91, 20.36)	0.0653
Congenital anomaly cohort (*N* = 197)^c^				
1	Race of pregnant person	White vs. Non-White	1.96 (0.90, 4.28)	0.0911

^a^Regular logistic regression was used for the periviable cohort model because the variance estimation for random site effect was zero. Language was not considered due to very small sample size of pregnant people who spoke a language other than English.

^b^Time from admission to delivery was used as a continuous variable in the model. The two values, 1 h and 3 h, were chosen to represent the early and later time in the 4-h period when drastic changes were seen in the probability of receiving consult between White and non-White pregnant people (Fig. 3).

^c^Mixed-effect logistic regression was used for the congenital cohort model. Time from admission to delivery was not considered to be clinically relevant in this cohort. Language was not able be tested as effect modifier since there was not enough data when stratified by the outcome and race. Prenatal care and insurance were dropped from the model due to their insignificance.

Resuscitation decision was not significantly associated with race and payor type. Resuscitation decision differed by receipt of neonatology consultation (*p* = 0.04) (Table 1). Among those who did not receive consultation, only 1 pregnant person opted for comfort care (5%). Conversely, among the 150 individuals who received consultation, 30 (20%) opted for limited resuscitation (*n* = 27) or comfort care (*n* = 3). There were no statistically significant differences related to timing of neonatology consultation and resuscitation decision.

A sensitivity analysis excluding the 3 periviable infants with congenital anomalies did not change the findings.

### Congenital anomaly cohort

The congenital anomaly cohort had 197 pregnant person-neonate dyads. Included people had a median age of 29.6 years and identified as primarily non-Hispanic White (79% White, 96% non-Hispanic) and English-speaking (95%) (Table 3). A slight majority had commercial insurance (55%). The median gestational age at delivery was 36 5/7 weeks. Most received consultation prior to delivery (79%) with the median gestational age at the time of consultation being 26 1/7 weeks. Most (85%) chose full resuscitation.

**Table 3:** Descriptive table of pregnant people by prenatal consult group among congenital anomaly cohort.

Variable	Overall (*N* = 197)	Did not receive a consult (*N* = 41)	Received a consult (*N* = 156)	*p* value^a^
**Demographics**				
Race				0.0045
White	155 (78.7%)	28 (18.1%)	127 (81.9%)	
Black or African American	30 (15.2%)	6 (20.0%)	24 (80.0%)	
Asian	1 (0.5%)		1 (100%)	
Native Hawaiian or Other Pacific Islander	1 (0.5%)		1 (100%)	
Multiracial/Unknown	10 (5.1%)	7 (70.0%)	3 (30.0%)	
Dichotomized race				0.0680
White	155 (78.7%)	28 (18.1%)	127 (81.9%)	
Non-White	42 (21.3%)	13 (31.0%)	29 (69.0%)	
Ethnicity				0.1588
Non-Hispanic	190 (96.4%)	38 (20.0%)	152 (80.0%)	
Hispanic	7 (3.6%)	3 (42.9%)	4 (57.1%)	
Preferred language				0.0204
English	188 (95.4%)	36 (19.1%)	152 (80.9%)	
Other	9 (4.6%)	5 (55.6%)	4 (44.4%)	
Age at delivery (years)				0.3896
Median (Q1, Q3)	29.6 (24.5, 33.2)	29.6 (27.2, 33.0)	29.6 (24.2, 33.4)	
Insurance payor type				0.6426
Medicaid or self-pay	88 (44.7%)	17 (19.3%)	71 (80.7%)	
Commercial	109 (55.3%)	24 (22.0%)	85 (78.0%)	
Community deprivation index				0.5524
Median (Q1, Q3)	0.3 (0.3, 0.4)	0.3 (0.3, 0.4)^b^	0.3 (0.3, 0.4)^c^	
**Prenatal care**				
Fertility treatment				0.6725
No	189 (95.9%)	39 (20.6%)	150 (79.4%)	
Yes	8 (4.1%)	2 (25.0%)	6 (75.0%)	
Prenatal care				0.2030
Good	182 (92.4%)	40 (22.0%)	142 (78.0%)	
Inadequate (None, Scant, or Late)	15 (7.6%)	1 (6.7%)	14 (93.3%)	
Birth hospital				0.0542
One level IV NICU	56 (28.4%)	7 (12.5%)	49 (87.5%)	
One academic level III NICU	99 (50.3%)	21 (21.2%)	78 (78.8%)	
One private level III NICU	39 (19.8%)	11 (28.2%)	28 (71.8%)	
Two level II NICUs	3 (1.5%)	2 (66.7%)	1 (33.3%)	
Gestational age at consult (weeks)				–
Median (Q1, Q3)	26.1 (22.9, 32.1)		26.1 (22.9, 32.1)	
Consultation location				–
Fetal Care Center (FCC)	110 (71.0%)		110 (100%)	
Inpatient	45 (29.0%)		45 (100%)	
**Infant anomaly**				
Periviability	3 (1.5%)	0 (0.0%)	3 (100%)	1.0000
Hydrops	44 (22.3%)	6 (13.6%)	38 (86.4%)	0.1834
Chromosomal	27 (13.7%)	7 (25.9%)	20 (74.1%)	0.4811
Fetal malformation	41 (20.8%)	2 (4.9%)	39 (95.1%)	0.0047
Renal	36 (18.3%)	0 (0.0%)	36 (100%)	0.0007
CNS	24 (12.2%)	3 (12.5%)	21 (87.5%)	0.4216
Cardiac	35 (17.8%)	26 (74.3%)	9 (25.7%)	<0.0001
Number of severe anomalies				1.0000
1	183 (92.9%)	38 (20.8%)	145 (79.2%)	
2	14 (7.1%)	3 (21.4%)	11 (78.6%)	
Number of severe anomaly categories				0.4369
1	187 (94.9%)	38 (20.3%)	149 (79.7%)	
2	10 (5.1%)	3 (30.0%)	7 (70.0%)	
**Outcomes**				
Gestational age at birth (weeks)				<0.0001
Median (Q1, Q3)	36.7 (33.7, 37.9)	38.0 (37.0, 39.0)	36.1 (33.0, 37.4)	
Timing: admission to consult (days)				–
Median (Q1, Q3)			−45.4 (−88.8, −1.3)	
Timing: consult to birth (days)				–
Median (Q1, Q3)			51.1 (11.1, 91.5)	
Decision for resuscitation				0.3149
Full active resuscitation		36 (21.4%)	132 (78.6%)	
Limited resuscitation			9 (100%)	
Comfort care		5 (25.0%)	15 (75.0%)	

^a^Wilcoxon rank sum test for continuous variables and Chi-square or Fisher’s exact test for categorical variables.

^b^*n* = 40 and ^c^*n* = 149 given inability to geocode available address information.

Diagnoses were grouped into categories: hydrops (22%), chromosomal anomalies (14%), central nervous system (CNS) anomalies (12%), cardiac anomalies (18%), renal anomalies (18%), and other fetal malformations (21%). A total of 7% had more than one severe anomaly, and 5% had anomalies in multiple diagnostic categories (e.g., alobar holoprosencephaly and Trisomy 13); 2% were delivered at a periviable gestational age.

In this cohort, White pregnant people received consultation more frequently than their non-White counterparts (82% vs. 69%, *p* = 0.07) (Table 3). When the 3 infants who were born in the periviable period were excluded, 82% of White pregnant people vs. 68% of non-White pregnant people received a consult (*p* = 0.05). Pregnant people who spoke a language other than English were less likely to receive a consult than those who spoke English (44% vs. 81%, *p* = 0.02). In bivariate analysis, we found no statistical differences between prenatal consultation and ethnicity of the pregnant person, payor type, prenatal care, or community material deprivation index. Of note, 14 of 15 pregnant people (93%) with inadequate prenatal care received prenatal consultation. In multivariable analysis, sample size did not allow for consideration of preferred language as an effect modifier. Language was initially included in the model as a covariate; however, it was not included in the final model due to insignificance (Supplementary Table 2). Only the race of the pregnant person remained in the model with site as a random effect (Table 2).

Among those who did not receive neonatology consultation, the majority (63%) delivered neonates with critical cardiac defects. This is consistent with our institutional practice; prenatal neonatology consultation does not typically occur for isolated cardiac anomalies. Rather, those patients are generally seen by a pediatric cardiologist. Among the remaining, 6 had hydrops (2 delivered preterm), 3 had Trisomy 13, 3 had Trisomy 18, 1 had triploidy, 1 had anencephaly, and 1 had pontocerebellar hypoplasia. In a sensitivity analysis excluding isolated cardiac anomalies, 93% of White vs. 79% of non-White pregnant people received neonatology consultation (*p* = 0.02).

There were no statistically significant differences in the timing of consultation or the location of the initial consult by race or payor type. Likewise, resuscitation decisions did not differ by race or payor type in the main or sensitivity analyses.

## Discussion

In this regional assessment of prenatal consultation and parental resuscitation decisions, we found previously unrecognized inequities in prenatal neonatology consultation for periviable births by the race, ethnicity, and payor type of the pregnant person. Among infants with congenital anomalies, inequities in consultation were found by language with potential inequities by race. Such findings likely indicate the effects of structural racism and social determinants on healthcare delivery [29–33].

While most pregnant people who delivered during the periviable period received prenatal consultation, pregnant people of non-White race had lower odds of receiving consultation. If non-White pregnant people had met with a neonatologist with the same frequency as their White peers, an additional 11 of the 85 non-White pregnant people would have received prenatal consultation, potentially influencing their peripartum course.

In the periviable cohort, access to care appeared to be a contributing factor. Pregnant people who did not receive consultation were more likely to have experienced inadequate prenatal care than those who received consultation, suggesting potential healthcare access difficulties. We also identified a sizeable difference in time from admission to delivery between the group of pregnant people who received consultation and those who did not. The rapid progression to delivery may have prevented the opportunity for prenatal counseling for many. We were unable to assess the impact of preferred language on consultation with few families speaking a language other than English in this cohort. It will be essential to explore factors influencing when pregnant people present for initial and subsequent prenatal care. This may include challenges related to transportation, childcare, language access, receipt of appropriate anticipatory guidance, and mistrust of the healthcare system.

However, a short interval from admission to delivery does not account for all the differences we uncovered. While 55% of those who received a consult did so in less than 6 h from admission, some pregnant people without consultations had admission-to-delivery durations of 7–32 h. Furthermore, there was a striking racial inequity among those who delivered within 1 h of presentation, with White pregnant people having nearly 7 times the odds of receiving a consultation.

Our findings add to existing data surrounding perinatal consultation practices. Feltman et al. examined periviability consultation practices at 6 centers [17]. While they found low overall rates of consultation (40%, 63%, and 72% of 498 pregnant people delivering at 22, 23, and 24 weeks, respectively), they did not find inequities by race. Like our findings, they noted pregnant people who received consultations had longer median admission-to-delivery intervals and a higher percentage of regular prenatal care than those who did not. There are limited data regarding consultation practices for fetuses with congenital anomalies. A retrospective population-level review of fetuses and infants with Trisomy 13 and Trisomy 18 (including lost or terminated pregnancies and stillborn infants) within our center demonstrated that only 37.9% of the families had a prenatal neonatology consult. Only 16.1% of these families were seen in our multidisciplinary Fetal Care Center (FCC) [18]. In our study, 76.9% of families with liveborn infants with Trisomy 13 and Trisomy 18 received a prenatal neonatology consultation; 55% of those consults occurred in our FCC.

In contrast to published findings on delivery room resuscitation practices [13, 14], we did not find differences in resuscitation decisions by race, with most families opting for full resuscitation. Feltman et al. also did not find racial differences in preferences for resuscitation [17]. However, within the NICU, prior work has documented racial inequities in redirection of care, the practice of opting to withdraw, withhold, or limit life-sustaining medical therapies; these conversations occurred less often for Black and Hispanic infants than for White and non-Hispanic infants [34]. A separate study suggested that fewer parents of Black infants may agree to physician-recommended limitations in care; however, this finding did not reach statistical significance [35].

The nature of a retrospective chart review limits our ability to ascertain decision-making regarding the choice and timing of neonatology consultation, the quality of the consultation with neonatology, and parental decision-making regarding resuscitation. Some pregnant people may not have been offered a neonatology consultation or resuscitation if the fetus was deemed previable or nonviable by the obstetrician. This work lays the necessary foundation for future studies, which could investigate potential drivers of inequities in referral patterns and further explore the quality of the consultation provided.

We recognize limitations regarding the identification of eligible patients. Some families who opt for comfort care may deliver at a level II hospital; we included 2 affiliated level II nurseries to address this concern. We limited our population to liveborn neonates for the feasibility of data abstraction and cohort identification. However, some families may opt to terminate the pregnancy.

Likewise, the total cohort of pregnant people of infants with severe congenital anomalies is likely affected by selection bias. Many such families are seen in our ambulatory FCC before delivery. Inequities in the care of this population may occur prior to referral to the FCC or may result from differential ability to complete an initial evaluation because of barriers with transportation, housing, childcare, or medical leave. Existing data demonstrates inequities in access to fetal care centers by race, ethnicity, and insurance payor type [36, 37]. Furthermore, given the sample size, we were underpowered to detect the observed difference between White vs. non-White groups in the main analysis cohort, limiting our ability to draw firm conclusions. Nonetheless, we found significant inequities in consultation practice by preferred language, and sensitivity analyses excluding periviable infants or those with cardiac anomalies demonstrated inequities by race.

Strengths of our study include its broad scope with consideration beyond periviability and our population-based, geographically defined regional care system. While some studies have investigated inequities in the care of periviable infants, our study is the first to expand this investigation to other high-mortality conditions, such as congenital anomalies, and to place a specific focus on caregiver resuscitation decisions. Moreover, the extent of the regional care that our institution provides allows our work to capture a population-level assessment of infants affected by high-mortality conditions.

## Conclusion

This regional assessment of perinatal care delivery found previously unrecognized inequities in prenatal neonatology consultation practices. Further work is needed to assess drivers of inequitable care delivery for this vulnerable population.

## Supplementary information

Supplemental Table 1

Supplemental Table 2

## Data Availability

The deidentified datasets generated during and/or analyzed during the current study are available from the corresponding author on reasonable request.

## Code availability

Programming code is generally considered proprietary information. If specific questions arise, these may be directed to the corresponding author.
