# Supplementary material for: Inequities in prenatal neonatology consultation in high-mortality neonatal populations
Source: J Perinatol. 2025 Aug 20;45(9):1198–206. doi: 10.1038/s41372-025-02377-z (PMC12431841; doi:10.1038/s41372-025-02377-z)
Supplement: Supplementary file 1 — Supplemental Table 1 [file 41372_2025_2377_MOESM1_ESM.docx]

Supplemental Table 1. ICD-9 and ICD-10 diagnostic codes for eligible infant conditions.

| **Diagnoses** | **ICD-10 Codes** | **ICD-9 Codes** |
| --- | --- | --- |
| Periviability (22.0 – 24.6 weeks gestation) | - P07 Disorders of newborn related to short gestation and low birth weight, not elsewhere classified   - P07.0 Extremely low birth weight newborn     - P07.00 …… unspecified weight     - P07.01 …… less than 500 grams     - P07.02 …… 500-749 grams     - P07.03 …… 750-999 grams   - P07.2 Extreme immaturity of newborn     - P07.20 …… unspecified weeks of gestation     - P07.21 …… gestational age less than 23 completed weeks     - P07.22 …… gestational age 23 completed weeks     - P07.23 …… gestational age 24 completed weeks | - 765.0 Disorders relating to extreme immaturity of infant   - 765.00 Extreme immaturity, unspecified [weight]   - 765.01 Extreme immaturity, less than 500 grams   - 765.02 Extreme immaturity, 500-749 grams   - 765.03 Extreme immaturity, 750-999 grams   OR   - 765.1 Disorders relating to other preterm infants    - 765.10 Other preterm infants, unspecified [weight]   - 765.11 Other preterm infants, less than 500 grams   - 765.12 Other preterm infants, 500-749 grams   - 765.13 Other preterm infants, 750-999 grams   OR   - 765.2 Weeks of gestation   - 765.20 Unspecified weeks of gestation   - 765.21 Less than 24 completed weeks of gestation   - 765.22 24 completed weeks of gestation   - 765.23 25-26 completed weeks of gestation   - 765.24 27-28 completed weeks of gestation |
| Hydrops | - P02.3 Newborn affected by placental transfusion syndromes - P83.2 Hydrops fetalis not due to hemolytic disease - P56 Hydrops fetalis due to hemolytic disease   - P56.0 Hydrops fetalis due to isoimmunization   - P56.9 Hydrops fetalis due to other and unspecified hemolytic disease     - P56.90 Hydrops fetalis due to unspecified hemolytic disease     - P56.99 Hydrops fetalis due to other hemolytic disease | - 773.3 Hydrops foetalis due to isoimmunisation - 778.0 Foetal hydrops - 778.0 Hydrops foetalis not from isoimmunisation - 653.7 Foetal hydrops - 653.70 Foetal hydrops causing disproportion |
| High-risk sacrococcygeal teratoma | - D48.0 Neoplasm of uncertain behavior of bone and articular cartilage | - 238.0 Neoplasm of uncertain behavior of bone and articular cartilage |
| Conjoined twins | - Q89.4 Conjoined twins | - 678.1 Fetal conjoined twins complicating pregnancy - 678.1 Fetal conjoined twins - 678.11 Foetal conjoined twins, delivered, with or without mention of antepartum condition - 678.11 Foetal conjoined twins complicating pregnancy with delivery - 678.10 Foetal conjoined twins affecting pregnancy - 678.13 Foetal conjoined twins affecting pregnancy, antepartum - 678.11 Foetal conjoined twins, delivered - 678.11 Foetal conjoined twins affecting pregnancy, delivered - 678.10 Foetal conjoined twins - 678.11 Foetal Siamese twins, delivered, with or without mention of antepartum condition - 678.10 Foetal conjoined twins complicating pregnancy - 678.10 Foetal Siamese twins - 759.4 Conjoined twins with two heads |
| Trisomy 13 | - Q91 Trisomy 18 and Trisomy 13   - Q91.4 Trisomy 13, nonmosaicism (meiotic nondisjunction)   - Q91.5 Trisomy 13, mosaicism (mitotic nondisjunction)   - Q91.6 Trisomy 13, translocation   - Q91.7 Trisomy 13, unspecified | - 7581. Patau’s syndrome |
| Trisomy 18 | - Q91 Trisomy 18 and Trisomy 13   - Q91.0 Trisomy 18, nonmosaicism (meiotic nondisjunction)   - Q91.1 Trisomy 18, mosaicism (mitotic nondisjunction)   - Q91.2 Trisomy 18, translocation   - Q91.3 Trisomy 18, unspecified | - 758.2 Edwards' syndrome |
| Trisomy 22 | - Q92 Other trisomies and partial trisomies of the autosomes, not elsewhere classified   - Q92.0 Whole chromosome trisomy, nonmosaicism (meiotic nondisjunction)   - Q92.1 Whole chromosome trisomy, mosaicism (mitotic nondisjunction)   - Q92.8 Other specified trisomies and partial trisomies of autosomes   - Q92.9 Trisomy and partial trisomy of autosomes, unspecified | - 758.0 Trisomy syndrome, 22 - 758.3 Trisomy 22 Syndrome - 758.3 Complete Trisomy 22 Syndrome |
| Triploidy | - Q92.7 Triploidy and polyploidy | - 758.9 Conditions due to anomaly of unspecified chromosome |
| Limb-Body Wall Complex | - Q74.9 Unspecified congenital malformation of limb(s) - Q79.59 Other congenital malformations of abdominal wall - Q67.8 Other congenital deformities of chest | - 759.89 Other specified congenital anomalies - 759.9 Congenital anomaly, unspecified - 756.79 Other congenital anomalies of abdominal wall - 755.9 Unspecified anomaly of unspecified limb - 754.89 Other specified nonteratogenic anomalies |
| Sirenomelia | - Q82.7 - Q89.8 Other specified congenital malformations | - 759.89 Other specified congenital anomalies - 759.9 Congenital anomaly, unspecified |
| Campomelic Dysplasia | - Q89.9 Congenital malformation, unspecified | - 759.89 Other specified congenital anomalies - 759.9 Congenital anomaly, unspecified |
| Thanatophoric Dysplasia | - Q77.1 Thanatophoric short stature | - 756.4 Chondrodystrophy - 756.59 Thanatophoric dysplasias |
| Short Rib Polydactyly Syndrome | - Q77.2 Short rib syndrome - Q87.89 Other specified congenital malformation syndromes, not elsewhere classified - Q69.9 Polydactyly, unspecified | - 756.3 Other anomalies of ribs and sternum - 759.89 Other specified congenital anomalies - 755.00 Polydactyly, unspecified digits |
| Osteogenesis Imperfecta (Severe Phenotypes) | - Q78.0 Osteogenesis imperfecta | - Specific code 756.51 Osteogenesis imperfecta - 520.5 Osteogenesis imperfecta with blue sclerae and dentinogenesis imperfecta |
| Severe Congenital Diaphragmatic Hernia (determined based on the o/e LHR, PPLV, TLV to be in the severe category) | - Q79 Congenital malformations of musculoskeletal system, not elsewhere classified   - Q79.0 Congenital diaphragmatic hernia   - Q79.1 Other congenital malformations of diaphragm | - V13.67 History of congenital diaphragmatic hernia - V13.68 Personal history of (corrected) congenital diaphragmatic hernia or other congenital diaphragm malformations - 756.6 Anomalies of diaphragm |
| Pentalogy of Cantrell | - Q28.1 Other malformations of precerebral vessels | - 747.89 Other specified anomalies of circulatory system |
| OEIS complex (omphalocele-exstrophy-imperforate anus-spinal defects) | - Q87.89 Other specified congenital malformation syndromes, not elsewhere classified   AND   - Q79.2 Exomphalos   AND   - Q64.12 Cloacal exstrophy of urinary bladder   AND   - Q76.49 Other congenital malformations of spine, not associated with scoliosis   AND   - Q42.3 Congenital absence, atresia and stenosis of anus without fistula | - 759.89 Other specified congenital anomalies   AND   - 553.1 Exomphalos   AND   - 753.5 Exstrophy of urinary bladder   AND   - 756.1 Congenital anomalies of spine   - 756.10 Anomaly of spine, unspecified   - 756.19 Other anomalies of spine   AND   - 751.2 Atresia and stenosis of large intestine, rectum, and anal canal |
| Bladder Outlet Obstruction | - N32.0 Bladder-neck obstruction | - 596.0 Bladder neck obstruction |
| Multicystic Dysplastic Kidneys with Anhydramnios | - Q61.4 Renal dysplasia | - 753.15 Renal dysplasia |
| Polycystic kidney disease | - Q61.1 Polycystic kidney, infantile type   - Q61.11 Cystic dilatation of collecting ducts   - Q61.19 Other polycystic kidney, infantile type - Q61.9 Cystic kidney disease, unspecified | - 753.1 cystic kidney disease   - 753.12 Polycystic kidney, unspecified type   - 753.13 Polycystic kidney, autosomal dominant   - 753.14 Polycystic kidney, autosomal recessive |
| Bilateral Renal Agenesis/Potter’s Sequence | - Q60.0 Renal agenesis, unilateral - Q60.6 Potter's syndrome | - 753.0 Renal agenesis and dysgenesis |
| Meckel Gruber Syndrome | - Q61.9 Cystic kidney disease, unspecified | - 753.10 Cystic kidney disease, unspecified |
| Encephalocele (giant encephalocele or encephalocele with brainstem involvement) | - Q01 Encephalocele   - Q01.0 Frontal encephalocele   - Q01.1 Nasofrontal encephalocele   - Q01.2 Occipital encephalocele   - Q01.8 Encephalocele of other site   - Q01.9 Encephalocele, unspecified | - 742.0 Encephalocele |
| Craniorachischisis | - Q00.1 Craniorachischisis | - 740.1 Craniorachischises - 740.1 Craniorachischisis |
| Exencephaly | - Q01.9 Encephalocele, unspecified | - 742.0 Encephalocele |
| Iniencephaly | - Q00.2 Iniencephaly | - 740.2 Iniencephaly |
| Anencephaly/Acrania | - Q00.0 Anencephaly | - 740.0 Anencephalus |
| Alobar Holoprosencephaly | - Q04.2 Holoprosencephaly | - 742.2 Congenital reduction deformities of brain |
| Lissencephaly | - Q04.3 Other reduction deformities of brain | - 759.89 Lissencephaly syndrome |
| Pontocerebellar hypoplasia | - Q04.3 Other reduction deformities of brain | - 742.2 Congenital reduction deformities of brain |
| Hydranencephaly | - Q04.3 Other reduction deformities of brain | - 742.3 Hydranencephalies |
| Vein of Galen Malformation | - Q28.3 Other malformations of cerebral vessels | - 747.81 Anomalies of cerebrovascular system |
| Acardia | - Q89.8 Other specified congenital malformations | - 759.89 Other specified congenital anomalies |
| Hypoplastic Left Heart Syndrome with intact/severely restrictive atrial septum | - Q23.4 Hypoplastic left heart syndrome | - 746.7 Hypoplastic left heart syndrome |
| Tetralogy of Fallot with pulmonary atresia and major aortopulmonary collateral arteries | - Q21.3 Tetralogy of Fallot   AND   - Q25.79 Other congenital malformations of pulmonary artery | - 745.2 Tetralogy of Fallot   AND   - 747.39 Other anomalies of pulmonary artery and pulmonary circulation |
| Harlequin fetus | - Q80.4 Harlequin fetus | - 757.1 Ichthyosis congenita |
