# Supplementary material for: Inequities in prenatal neonatology consultation in high-mortality neonatal populations
Source: J Perinatol. 2025 Aug 20;45(9):1198–206. doi: 10.1038/s41372-025-02377-z (PMC12431841; doi:10.1038/s41372-025-02377-z)
Supplement: Supplementary file 2 — Supplemental Table 2 [file 41372_2025_2377_MOESM2_ESM.docx]

Supplemental Table 2. Multivariable modeling investigating factors affecting probability of prenatal consult for pregnant people in the analysis cohorts.

| **Model** | **Effect** | **Contrast** | **Odds Ratio** | **P value** |
| --- | --- | --- | --- | --- |
| **Periviable Cohort (N=172)*** | | | | |
| 1 | Race of pregnant person | White vs. non-White | - | 0.0092 |
|  | Time from admission to delivery (hr) |  | - | 0.0096 |
|  | Time from admission to delivery by Race of pregnant person interaction | White vs. Non-White \| Time from admission to delivery** =1 hour | 6.88 (1.38, 34.20) | 0.0403 |
|  |  | White vs. Non-White \| Time from admission to delivery** =3 hours | 2.44 (0.51, 11.76) |  |
|  |  | 3 hours vs. 1 hour \| Race=White | 1.15 (0.97, 1.36) |  |
|  |  | 3 hours vs. 1 hour \| Race=Non-White | 3.24 (1.22, 8.58) |  |
|  | Insurance | Commercial vs. Medicaid/Self-pay | 4.31 (0.91, 20.36) | 0.0653 |
|  |  |  |  |  |
| 2 | Race of pregnant person | White vs. Non-White | 2.15 (0.56, 8.24) | 0.2618 |
|  | Insurance | Commercial vs. Medicaid/Self-pay | 0.22 (0.05, 0.97) | 0.0103 |
|  | Time from admission to delivery (hr) | 1 hour increase | 1.19 (1.04, 1.37) | 0.0461 |
|  |  |  |  |  |
| 3 | Race of pregnant person | White vs. Non-White \| Time from admission to delivery** =1 hour | 8.41 (1.41, 50.26) | 0.0099 |
|  |  | White vs. Non-White \| Time from admission to delivery** =3 hours | 2.97 (0.52, 17.08) |  |
|  | Time from admission to delivery (hr) | 3 hours vs. 1 hour \| Race=White | 1.15 (0.97, 1.37) | 0.0100 |
|  |  | 3 hours vs. 1 hour \| Race=Non-White | 3.26 (1.21, 8.77) |  |
|  | Time from admission to delivery by race of pregnant person interaction |  |  | 0.0422 |
|  | Insurance | Commercial vs. Medicaid/Self-pay\| Time from admission to delivery** =1 hour | 4.35 (0.87, 21.66) | 0.0727 |
|  |  | Commercial vs. Medicaid/Self-pay\| Time from admission to delivery** =3 hour | 4.35 (0.87, 21.66) |  |
|  | Time from admission to delivery by insurance interaction |  |  | 0.5932 |
| **Congenital Anomaly Cohort (N=197)***** | | | | |
| 1 | Race of pregnant person | White vs. Non-White | 1.96 (0.90, 4.28) | 0.0911 |
|  |  |  |  |  |
| 2 | Race of pregnant person | White vs. Non-White | 1.64 (0.72, 3.74) | 0.2383 |
|  | Language | English vs. Non-English | 4.01 (0.98, 17.05) | 0.0542 |
| *Regular logistic regression was used for the periviable cohort model because the variance estimation for random site effect was zero. Language was not considered due to very small sample size in the non-English subgroup.  **Time from admission to delivery was used as a continuous variable in the model. The two values, 1 hour and 3 hours, were chosen to represent the early and later time in the 4-hour period when drastic changes were seen in the probability of receiving consult between the two races.  ***Mixed-effect logistic regression was used for the congenital cohort model. Time from admission to delivery was not considered to be clinically relevant in this cohort. Language was not able to be tested as effect modifier since there was not enough data when stratified by the outcome and race. Instead, it was included in model 2 as a covariate; however, the wide confidence interval which indicates that this variable is not reliable, and the parameter estimate is not stable. Thus, it was omitted from the final model. Prenatal care and insurance were dropped from the model due to their insignificance. | | | | |
